# Validation the role of desmocollin-2 in osteosarcoma based on single cell and bulk RNA seq and experimental analyses

**DOI:** 10.7150/jca.87411

**Published:** 2023-08-21

**Authors:** Jiaxing Zeng, Yu Sun, Yunan Man, Haijun Tang, Long Xie, Maolin He

**Affiliations:** 1 Department of Spinal Surgery, The First Affiliated Hospital of Guangxi Medical University, Nanning, 530021, Guangxi, China.; 2 Department of Traumatic Surgery & Microsurgery & Hand Surgery, Guangxi Zhuang Autonomous Region People's Hospital, Nanning, 530021, Guangxi, China.; 3 Department of Orthopaedics, The Affiliated Yuebei People's Hospital of Shantou University Medical College, Shaoguan, Guangdong Province, China.

**Keywords:** osteosarcoma, DSC2, intercellular adhesion, biological function, molecular mechanism

## Abstract

**Background:** The aetiology of osteosarcoma (OS) remains unclear. Desmocollin-2 (DSC2) mediates intercellular adhesion and is involved in tumour progression. Therefore, we aim to investigate the potential role of DSC2 in OS.

**Methods:** We analyzed the expression, prognostic value and immune infiltration of DSC2 in OS via single cell and bulk RNA seq data. Besides, the expression and function of DSC2 in OS were further verified by in vitro experiment.

**Results:** We preliminarily determined that DSC2 was high expressed in OS, which was a risk factor for survival and had a strong relationship with immune cell infiltration. What's more, in vitro experiments also demonstrated that DSC2 was high expressed in OS cells, and silencing DSC2 would suppress proliferation, migration and invasion of OS cells.

**Conclusions:** DSC2 may serve as an oncogene, which exerts a crucial role in tumor progression, predicting prognosis and immune cell infiltration in OS.

## Introduction

Osteosarcoma (OS), a malignant bone tumor derived from bone marrow mesenchymal stem cells^1^, ^2^, primarily affects children and teenager^3^. At present, the main treatment methods for OS included surgical resection combined with neoadjuvant chemotherapy^4^, as well as radiotherapy, molecular targeted therapy^5^, ^6^. However, the prognosis of OS is still unsatisfied with survival rate less than 70%. The reason is that the pathogenesis and metastasis mechanism of OS are extremly complex and there is no effective biomarker to predict the prognosis^7^. Therefore, it is of great significance to explore the new biomarkers to improve the treatment and prognosis of OS.

In recent years, a growing number of studies demonstrated that intercellular adhesion dysfunction plays an important role in the development of tumor. Loss of adhesion within the primary tumor provides an opportunity for individual cancer cells to escape from the lymphatic and circulatory system^8^. Desmosome is essential for intercellular adhesion, which mediates cell-cell communication and intracellular signaling^9^-^12^ and maintains the structural integrity of multicellular organisms. Desmosome contains desmosome cadherin that includes desmoglein (DSG) and desmocollin (DSC). DSC2, a member of the DSC family, is extensively expressed in both healthy and malignant tissues^13^. Some studies showed that DSC2 was overexpressed in the prostate cancer^14^, breast cancer^15^ and lung cancer tissues^16^. Recent evidence has demonstrated that Mutations and abnormal expression of DSC2 often lead to dysfunction of desmosome, which further cause the metastasis and progression of cancer cell in some malignant solid tumor. However, the biological function and underlying molecular mechanism of DSC2 in OS remains unclear. Considering that OS is also a solid tumor, we suspected that DSC2 may exert a crucial role in the proliferation and metastasis of OS.

In this study, we analyzed the expression, prognostic value and immune infiltration of DSC2 in OS via single cell and bulk RNA seq data. What's more, the expression and function of DSC2 in OS were further verified by in vitro experiment.

## Materials and methods

### Source of public databases

Single cell RNA seq data of OS and normal bone tissue was obtained from Gene Expression Omnibus (GEO) database with access number of GSE162454 and GSE169396, respectively. Bulk RNA seq data and clinical message of 85 OS patients was obtained from Therapeutically Applicable Research to Generate Effective Treatments (TARGET) database (https://ocg.cancer.gov/programs/target). The detailed criteria for the inclusion and exclusion were as following. The inclusion criteria are: 1) patients with age under 45 years; 2) patients with complete follow-up data; 3) patients that DSC2 can be detected. The exclusion criteria is: 1) Follow-up time less than 6 months. The Association between DSC2 expression and clinicopathological parameter in OS samples based on TARGET database was showed in Table 1. 12 mRNA expression datasets of DSC2 in OS tissues and cell lines were obtained from Gene Expression Omnibus (GEO) database using the MESH terms “osteosarcoma” OR “osteosarcomas” AND “Homo sapiens”. The access numbers were as following: GSE12865, GSE11414, GSE14359, GSE19276, GSE33383, GSE36001, GSE39262, GSE42352, and GSE68391. The single nucleotide mutation dataset was downloaded from GDC (https://portal.gdc.cancer.gov/). Finally, we collected the normalized mRNA expression profiles and corresponding clinical information from the Cancer Genome Atlas (TCGA) (https://portal.gdc.cancer.gov/) Pan-Cancer (n=10535) dataset.

### Single-cell sequencing data processing

Two single cell datasets were combined by the "seurat" R package. Low-quality cells were filtered with criteria of nFeature_RNA>300, nFeature_RNA<4500, and per cent.mt<10. Besides, the batch effect was removed from the data after quality control by using the "harmony" package. Subsequently, we performed t-SNE algorithms to explore and visualize the cluster classification of the entire cell sample. Next, in the cell definition, we selected ALPL, RUNX2 and IBSP to define osteoblast-like cells (OB cells). The expression abundance of DSC2 in OB cells between the normal and OS cohorts were compared.

### Identifying the relationship between DSC2 and prognosis

The prognosis value of DSC2 was investigated by "survminer" R packages base on the TARGET cohort. The group method of Kaplan-Meier survival analysis was based on optimal cutoff values. Besides, different clinical characteristic was also added to the survival analysis. Finally, a nomogram including clinical characteristic and DSC2 was established using “rms” package.

### Identification of co-expressed genes correlated to DSC2

The genes co-expressed with DSC2 in TARGET dataset were identified on the basis of Pearson's correlation. Genes with r ≥0.78 and *p*< 0.001were regarded as co-expressed genes correlated to DSC2.

### Functional annotation of co-expressed genes correlated to DSC2

Gene Ontology (GO) functions and Kyoto Encyclopedia of Genes and Genomes (KEGG) pathways enrichment analysis of the DSC2 co-expressed genes were conducted with "clusterProfiler" R package. GO annotations were obtained from the genome-wide annotation package released by the Bioconductor project (org.Hs.eg.db). KEGG annotations were obtained from querying the latest online KEGG database. The criterion for significant enrichment was P value < 0.05.

### Demonstrating the DSC2-associated tumor immune landscape

Firstly, CIBERSORT algorithm was applied to identify the infiltrating fraction of immune cells based on OS transcriptome expression profiling data from the TARGET database. Meanwhile, correlation analysis was used to explore the correlation between the expression of DSC2 and immune cells. Finally, immune checkpoint gene and expression matrix data were extracted and correlation analysis was employed to investigate whether DSC2 was associated with immune checkpoint gene.

### Drug sensitivity analysis

Genes are central hubs in the mechanism of drug action. Therefore, the search for DSC2-sensitive drugs is important for treatment. In order to predict the association of drug sensitivity and the expression level of DSC2, “pRRophetic” package was used.

### Pan-cancer analysis

In order to profile the landscape of DSC2 mutations in multiple cancers, the single nucleotide mutation dataset was downloaded. Besides, we also investigated the expression of DSC2 in pan-cancers using UCSC Xena database.

### Cell culture

The human osteoblast cell line hFOB1.19 and the human OS cell lines U2OS, Saos2, 143B, HOS were purchased from the ATCC. The 143B cells were grown in medium 1640 (Gibco, USA), Saos2 cells in McCoy's 5A medium (Gibco, USA), and HOS and U2OS lines were cultivated in DMEM (Gibco, USA). All OS cell lines were maintained at 37°C in conditions containing 1% penicillin and streptomycin (Gibco, USA) and 10% fetal bovine serum (FBS, EXCELL, USA). hFOB1.19 cells were grown in DMEM including 10% FBS, 1% streptomycin/penicillin, and 0.3 mg/mL G418 (Gibco, USA) at 33.5°C.

### Cell transfection

SiRNA was used to knock down the DSC2 gene in U2OS and Saos2 cells. Three siRNA (siRNA-1: GGUUCAGUCUAUACAACAAdTdT, siRNA2: GGAGAGUUCUAC-UUCAGAAdTdT, siRNA-3: GGUCUACAGACAACUUCAAdTdT were purchased from hippo bio CO.,Ltd. (Zhejiang, China). Lipofectamine 2000(Lipo 2000) transfection reagent was bought from Thermo Fishner.

### qRT-PCR

Total RNA was extracted using TRIzol (MRC, China) and reverse-transcribed to cDNA byGoTaq® qPCR Master Mix (Promega, USA). The primer sequences for qRT-PCR were as follows: β-catin: 5'-CTCCATCCTGGCCTCGCTGT-3'(F) and 5'-ACTAAGTCATAGTCCGCCTAGA-3'(R); DSC2: 5'-TAACGCACTTAGGATTTGTCG-3'(F) and 5'-TACAGGAGAAATAAGGAGCAGC-3'(R). The corresponding expression of DSC2 was calculated utilizing the 2^-△△Ct^ technique with β-actin as the internal reference. The experiment was repeated thrice.

### Western blotting

The protein was extracted according to the RIPA buffer operating manual from the cell and the concentration was calculated by BCA method. Then, identical amounts of protein were fractionated using 10% SDS-PAGE and electroblotted onto PVDF films. Three times rinsed before overnight incubation with DSC2 (purchased from Abcam, Ab95967, dilution ratio: 1:2000) at 4 °C. The membrane was then washed and cultured for one hour with the secondary antibody (goat anti-rabbit IgG1:10,000).

### Proliferation assay

A Cell Counting kit-8 (CCK-8) (Everbright lnc., Sayreville, NJ, USA) was employed to measure cell proliferation of U2OS and Saos2. Cells were inoculated on 96-well plates with 1×10^4^ cells/well. According to the CCK-8 kit instructions, 10μL CCK-8 was added to each well and then cultured for 24h, 48h, 72h and 96h. Finally, the optical density was detected by an enzyme labeling instrument with 450nm wavelength. Each experiment was repeated three times.

### Cell apoptosis assay

Cell was transferred into 6-well plates at 2×10^5^ cells/well and incubated for 24h. The apoptosis of U2OS and Saos2 was assessed via the Annexin V-FITC/PI Kit (Life Technologies, Carlsbad, CA, USA), according to the manufacture's instruction, detected on a BD FACSCALIBUR flow cytometer.

### Wound healing assay

The migratory ability was measured by wound healing assay. A 6-well plate was cultivated with U2OS and Saos2 cell. Using a sterile 1000μL pipette tip to formulate a scratch, and then captured images at 0h and 24h.

### Transwell assay

Using Transwell method to evaluate the invasion capacity of U2OS and Saos2 cell. The cell was fostered in serum-free medium for 4-6h before this experiment and then transplanted to a Transwell chamber. Subsequently, 600μL of complete medium was supplemented to the lower chamber, and fostered for 24h, fixed in 4% paraformaldehyde for 20 minutes, then stained crystal violet.

### Statistical analysis

R software version 4.1.0 (http://www.R-project.org) was used to analyze and generate the data of bioinformatics. P < 0.05 was the statistically regarded significant and the "p.ucdilg" R function was used to adjust the P values for multiple analyses. SPSS Statistics 23.0 was used to analyze the experiment data, and the difference in the expression levels of DSC2 between the OS and control samples was calculated utilizing Student's t-test. Un paired Wilcoxon rank sum test was used to test the difference between two samples, and Kruskal-Wallis test to multiple groups of samples.

## Results

### DSC2 is upregulated in OS

**Figure 1:**
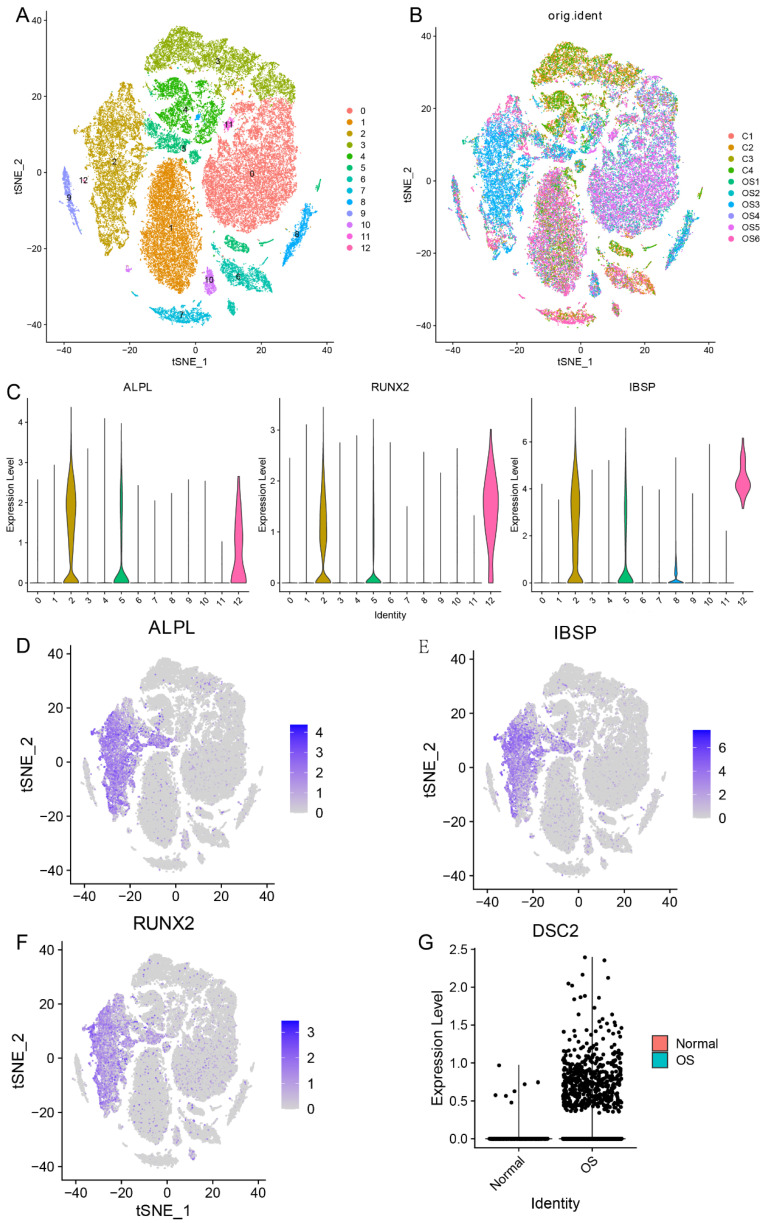
** Single cell RNA sequencing data landscape.** (A) 51599 cells were obtained and could be divided into 13 clusters. (B)Distribution of cells from 10 samples in 13cell clusters. (C) Cluster 2 was identified as OB cells for the specific high expression of ALPL, RUNX2 and IBSP. Distribution of ALPL(D), RUNX2(E) and IBSP(F) in cell clusters. (G) Differential expression of DSC2 in normal and OS cells.

Firstly, in order to compare the expression level of DSC2 between normal osteoblasts cells (OB cells) and tumor cells, we conducted single cell RNA seq analysis. After quality control, 51599 cells were obtained and could be divided into 13 clusters (**Figure 1A and Figure 1B**). Cluster 2 was identified as OB cells for the specific high expression of marker gene of osteoblasts (ALPL, RUNX2 and IBSP) (**Figure 1C-F**). After comparing, we found that the expression level of DSC2 of OB cells from OS tissue was significantly higher than that from normal bone tissue (**Figure 1G**).

**Figure 2:**
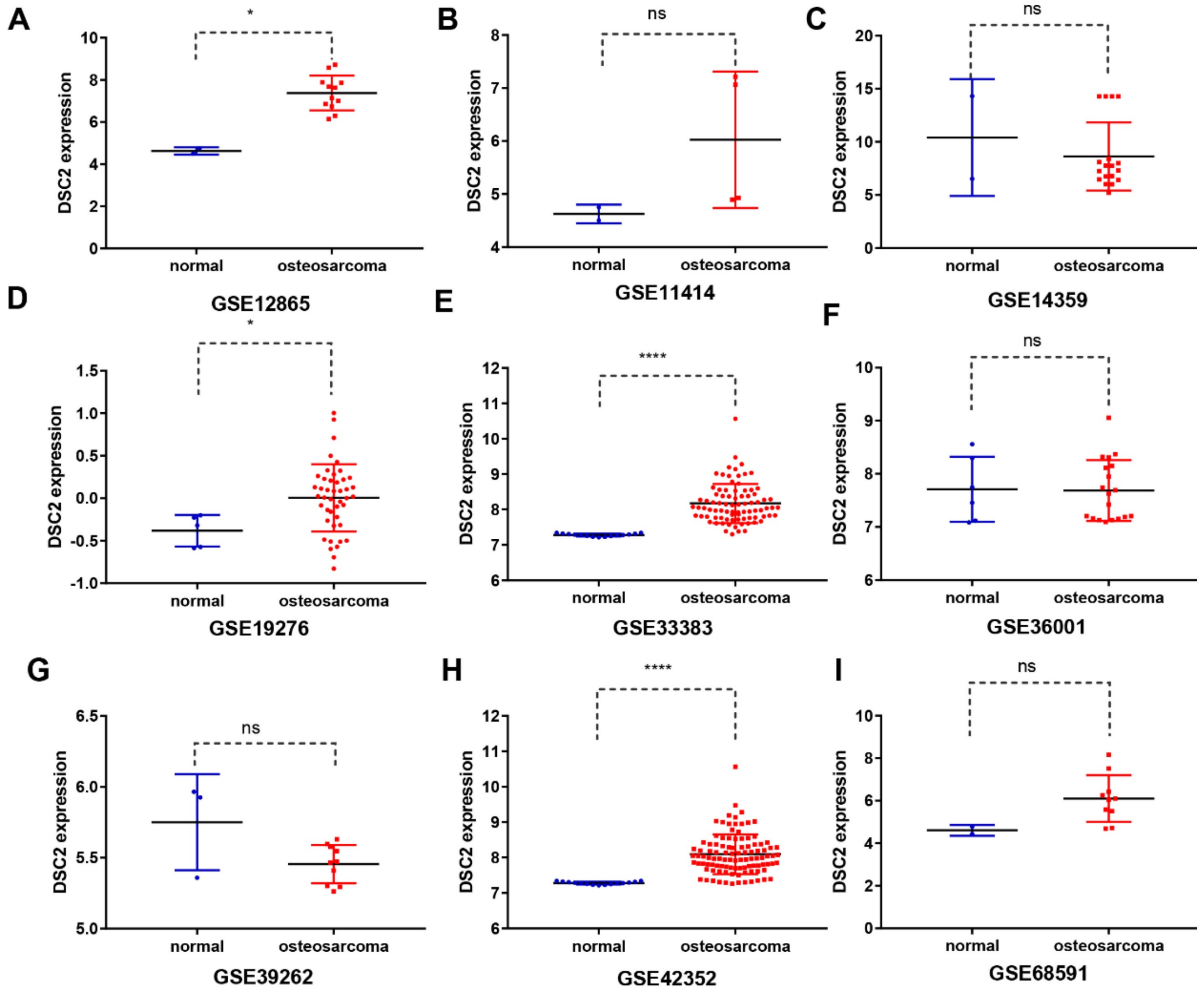
** DSC2 scatter plot based on mRNA expression datasets of OS in the GEO database.** The expression level of DSC2 in 12 OS mRNA expression datasets derived from GEO database was shown as follows: (A) GSE12865. (B) GSE11414. (C) GSE14359. (D) GSE19276. (E) GSE33383. (F) GSE36001. (G) GSE39262. (H) GSE42352. (I)GSE68591. (J) GSE87624. (K) GSE99671. (L) GSE126209. The mRNA of DSC2 in OS was up-regulated on the whole, compared with the matched non-OS samples.

Subsequently, the expression level of DSC2 in bulk RNA seq was also analyzed. We screened 9 OS mRNA expression datasets in the present study. DSC2 was increased in the OS compared to the control groups in some datasets, including GSE12865 (**Figure 2A**), GSE19276 (**Figure 2D**), GSE33383(**Figure 2E**) and GSE42352 (**Figure 2H**). The expression level of DSC2 did not differ significantly among other groups of GSE11414 (**Figure 2B**), GSE14359 (**Figure 2C**), GSE36001 (**Figure 2F**), GSE39262 (**Figure 2G**), GSE68591 (**Figure 2I**).

**Figure 3:**
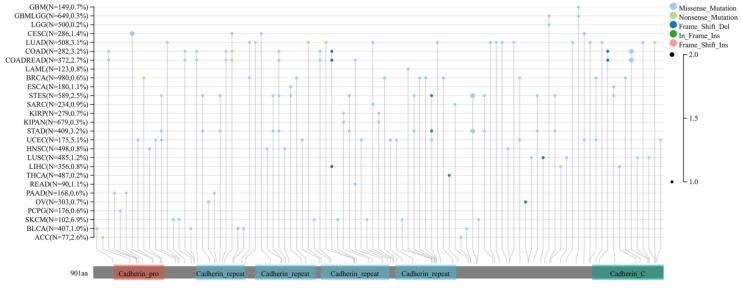
** Missense mutations of DSC2.** DSC2 was displayed a high frequency of gene mutations in some cancers, and the most common mutations in cancers were missense mutations. It suggests that DSC2 may act as a key oncogene for some cancers.

**Figure 4:**
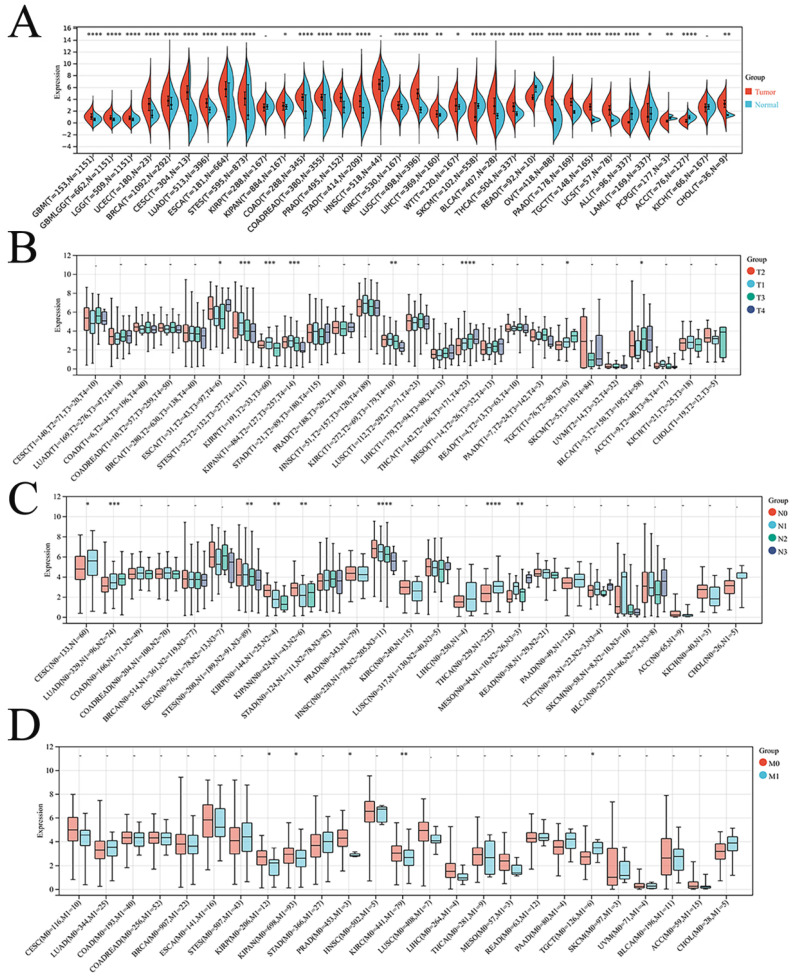
** Correlation between DSC2 expression and clinical stage of cancers.** (A) DSC2 expression in different cancers. (B) Relationship between DSC2 expression and T stage of cancers. (C) Relationship between DSC2 expression and N stage of cancers. (D) Relationship between DSC2 expression and M stage of cancers. TCGA Pan-Cancer datasets analysis revealed that DSC2 was differentially expressed in different cancers and involved in tumor progression.

Finally, the expression of DSC2 in pan-cancer was explored. As showed in **Figure 3**, DSC2 was displayed missense mutations in some cancers such as endometrial cancer and colon cancer, which indicated that DSC2 may be involved in the progression of cancers. On the other hand, based on the TCGA Pan-Cancer dataset, DSC2 was upregulated in most cancers including lung adenocarcinoma, breast invasive carcinoma, and so on (**Figure 4A**). Besides, DSC2 was differentially expressed in TNM stage of different cancers (**Figure 4B-D**).

To sum up, these results demonstrated that DSC2 is highly expressed in OS and some other malignant tumor, which released that DSC2 may play a key role in tumorigenesis and disease progression in OS via some molecular mechanisms.

### Effect of DSC2 on OS prognosis

**Figure 5:**
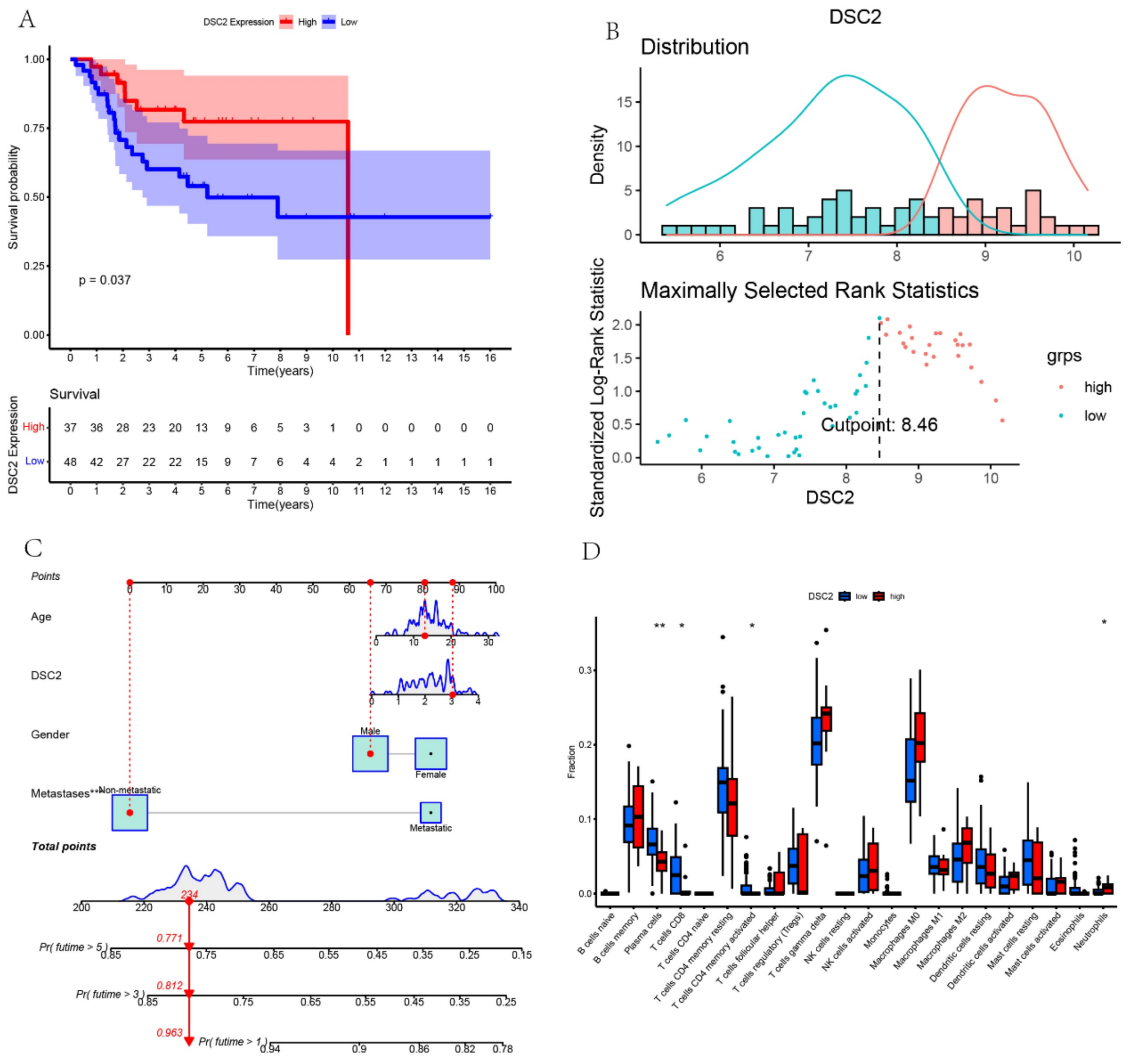
** Bulk RNA sequencing data landscape.** (A) Kaplan-Meier analysis of DSC2 associated with count values. (B)Identification of the optimal cutoff value for the count values. (C) The nomogram for predicting the prognosis of OS. (D) Differences in immune cell infiltration grouped by different DSC2 expression levels.

According to the optimal cutoff value of 8.46, patients in TARGET dataset were divided into two groups: low DSC2 expression group (n = 48) and high DSC2 expression group (n = 37). Kaplan-Meier analysis also showed a more advantageous overall survival rate in the low DSC2 expression group (**Figure 5A and 5B**). Meanwhile, the prognostic significance was still valid when using fpkm values (**Figure S1A and S1B**). **Figure S1C** showed the relationship among DSC2 expression and clinical phenotypes. Finally, the nomogram was established to further predict the prognosis of OS (**Figure 5C**).

### Immune landscapes associated with DSC2

**Figure 6:**
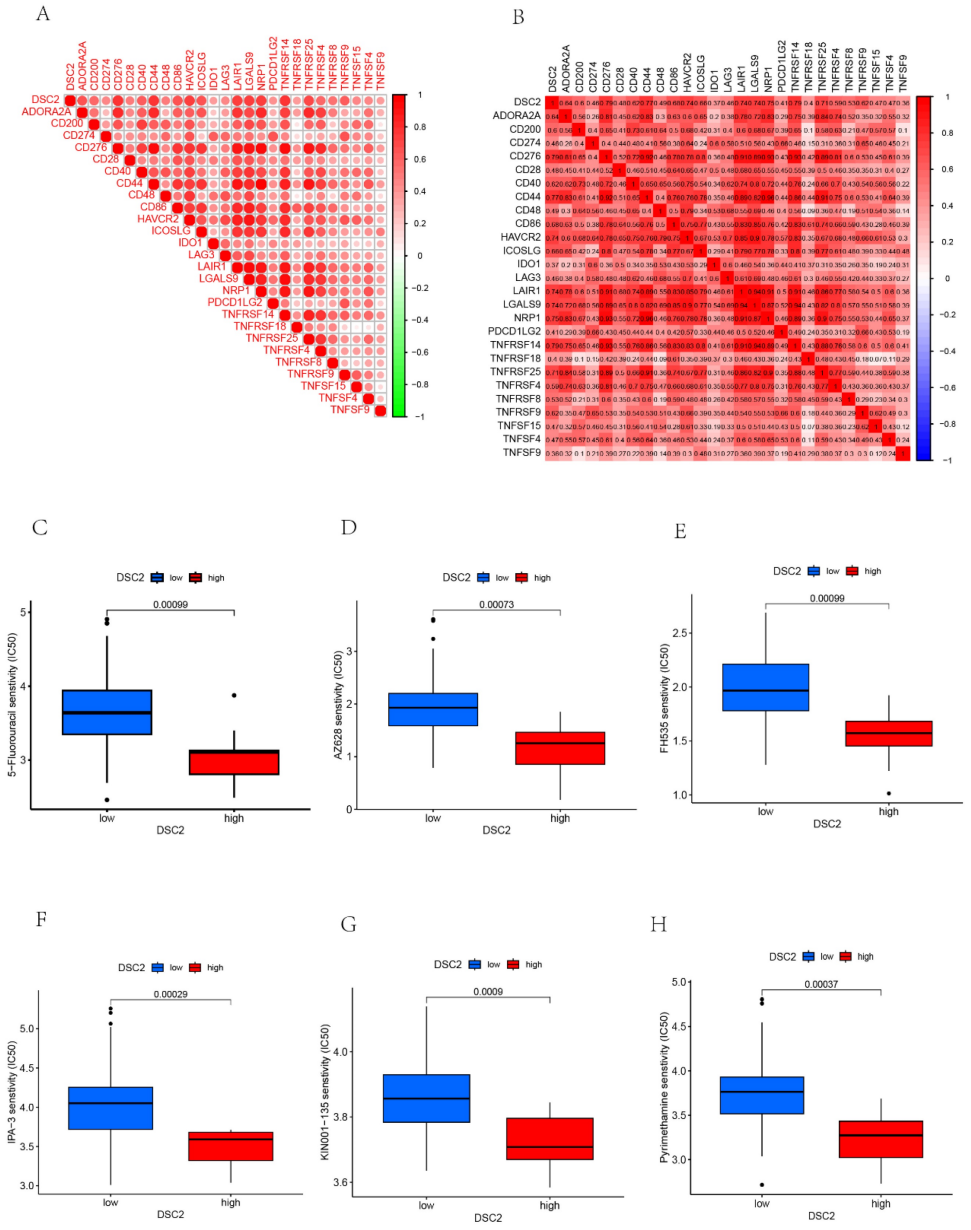
** DSC2-related immune response and drug sensitivity assays.** 26 immune checkpoint genes positively associated with DSC2. (B) Heat map of the correlation between each of the 26 immune checkpoint genes. Six drugs such as 5-Fluorouracil (C), AZ628 (D), FH535(E), IPA-3 (F), KIN001-135 (G), Pyrimethamine (H)had a significant sensitivity to DSC2.

As DSC2 is closely associated with osteosarcoma, we were concerned about the immune landscape associated with DSC2. We found a positive and statistically significant correlation between DSC2 and Plasma cells, CD8 T cells, CD4 memory activated T cells, and Neutrophils (**Figure 5D**). Next, six immune cell types, including Neutrophils, M2 Macrophages, Eosinophils, resting Dendritic cells, CD8 T cells and CD4 memory activated T cells were strongly and significantly related to DSC2 (**Figure S1D**). What's more, low DSC2 expression possessed more positive prognosis, so we generated interest in DSC2 on immune response. 26 immune checkpoint genes were found to have a statistically significant positive correlation with DSC2 by person correlation test (**Figure 6A and 6B**). In brief, these results suggest that DSC2 may be related to the immune invasion and the distribution of immune checkpoints in OS.

### Drugs sensitivity of DSC2

DSC2 may be an important target for the action of susceptibility drugs, and the screening of susceptibility drugs may offer new possibilities for drug treatment strategies. Six drugs such as 5-Fluorouracil, AZ628, FH535, IPA-3, KIN001-135, Pyrimethamine had a significant sensitivity to DSC2. The low DSC2 expressing group was more sensitive to these drugs (**Figure 6C-H**), indicating that DSC2 could be used as a target to these drugs.

### Identification of GO and KEGG pathway enrichment analysis associated with co-expressed genes of DSC2

**Figure 7:**
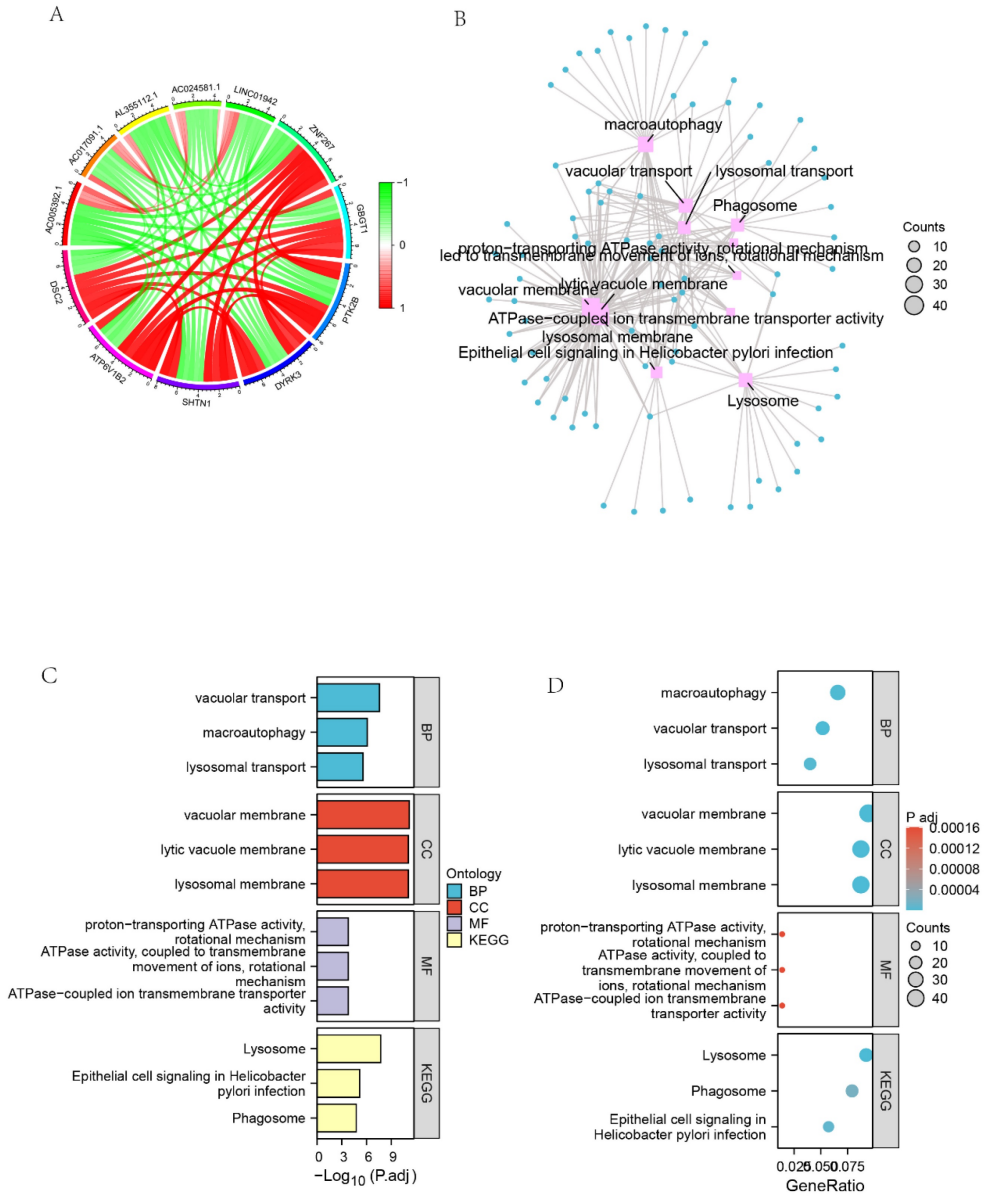
** Identification of GO and KEGG pathway enrichment analysis associated with co-expressed genes of DSC2.** (A) Circle diagram of the 11 most closely related genes to DSC2. (B) Network diagram of GO enrichment analysis of DSC2-related genes. (C) Histogram of GO enrichment analysis of DSC2-related genes. (D) Bubble plot of GO enrichment analysis of DSC2-related genes.

We screened 27,161 genes co-expressed with DSC2 from the TARGET dataset. **Figure 7A** showed the 11 genes that most strongly associated with DSC2. Furthermore, GO enrichment analysis of 449 co-expressed genes that met the screening criteria r > 0.78 & q<0.001 showed high enrichment for biological processes (BP) such as vacuolar transport, macroautophagy and lysosomal transport. Meanwhile, these genes mainly concentrated in the cellular components (CC) such as vacuolar membrane, lytic vacuole membrane and lysosomal membrane. The KEGG analysis indicated that the co-expressed genes were significantly associated with lysosome, epithelial cell signaling in Helicobacterpylori infection and phagosome pathways (**Figure 7 B-D**).

### Expression of DSC2 in OS cells

**Figure 8:**
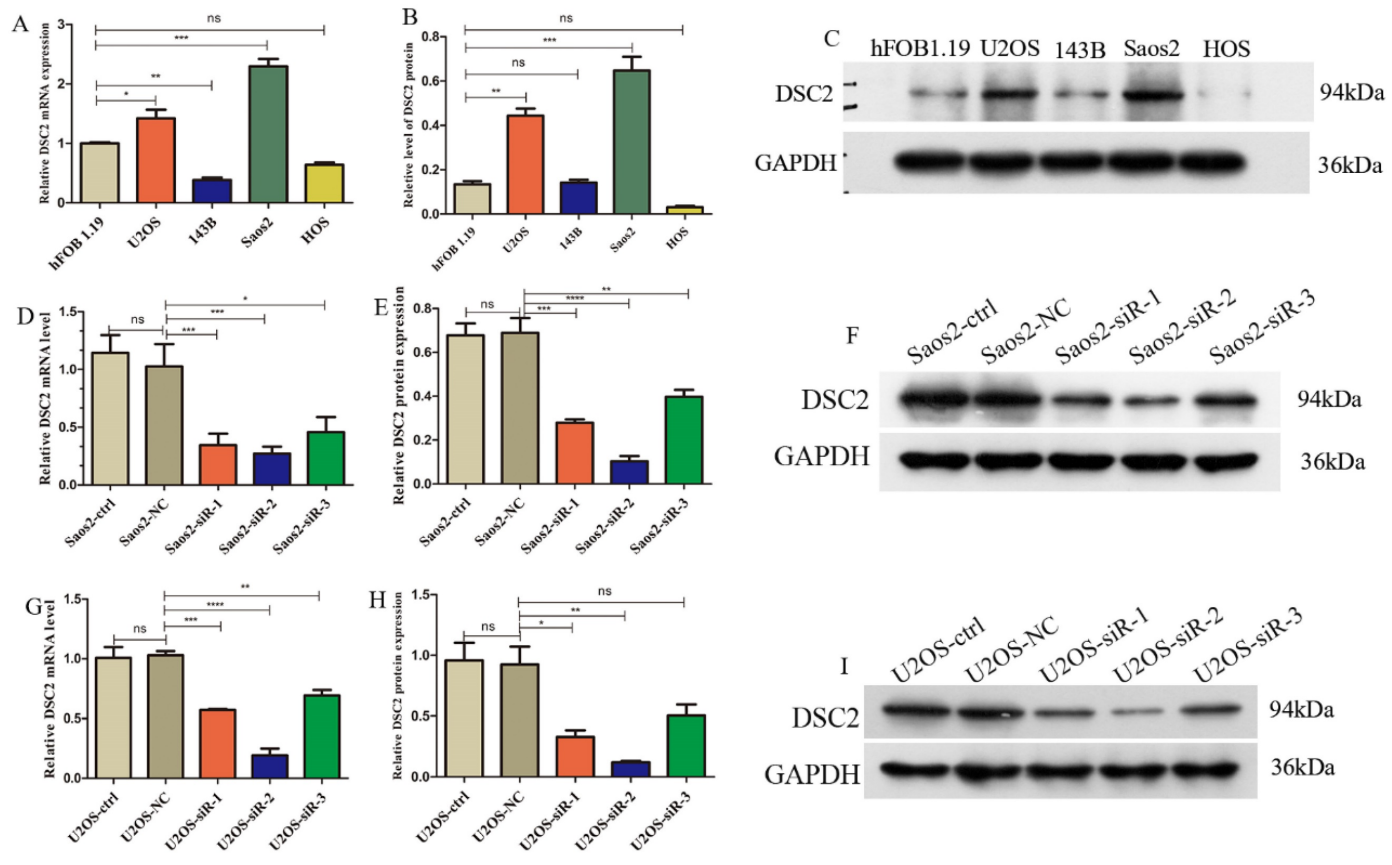
** Relative expression of mRNA and protein of DSC2 in OS cell lines and the silencing efficiency of DSC2 in U2OS and Saos2cell lines.** (A) mRNA expression level of DSC2 in osteosarcoma cell lines. (B) Protein expression level of DSC2 in osteosarcoma cell lines. (C) The protein expression of DSC2 in osteosarcoma cell lines was verified by WB. (D) Relative mRNA expression in Saos2 cells after DSC2 gene knockdown. (E) Relative protein expression after DSC2 knockdown in Saos2 cells. (F) WB validation results after DSC2 knockdown of Saos2. (G) Relative mRNA expression after knocking down DSC2 gene in U2OS cells. (H) Relative protein expression after knocking down DSC2 in U2OS cells. (I) WB validation results of U2OS after DSC2 knockdown. QRT-PCR and WB were performed to quantify the expression of DSC2 genes and protein in OS cell lines(U2OS, 143B, Saos2 and HOS) and normal osteoblast cell lines (hFOB1.19), and GAPDH was utilized as the internal control of WB assay. After DSC2 was silenced by siRNA in U2OS and Saos2, the silencing effect was assessed in comparison with the negative control (NC)siRNA group (statistical significance is indicated by ns, no significance; * P < 0.05; ** P < 0.01; *** P < 0.001; **** P < 0.0001).

DSC2 mRNA and protein expression levels in U2OS, Saos2,143B, HOS cell lines were validated by qRT-PCR and WB. Compared with the normal osteoblast cell line hFOB 1.19, the mRNA and protein expression of DSC2 was significantly up-regulated in U2OS and Saos2 cell lines (**Figure 8 A-C**).

Subsequently, we used siRNA to inhibit DSC2 in U2OS and Saos2 cells before measuring DSC2 mRNA and protein levels to determine silencing efficiency. From the results (**Figure 8 D-I**), siRNA-2 was the optimal site in the knockout expression, therefore, we chose siRNA-2 site in the next study.

### Effect of DSC2 on OS cell proliferation, apoptosis, migration and invasion

**Figure 9:**
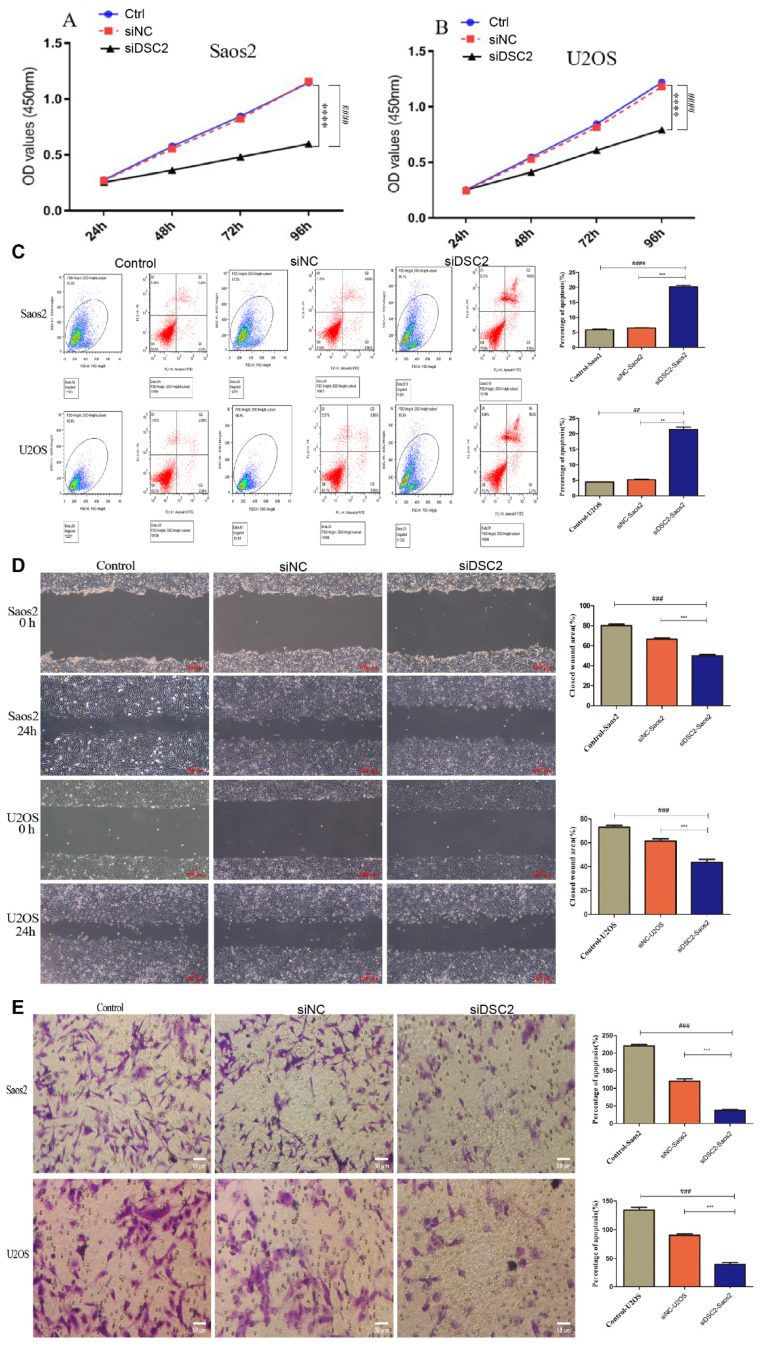
** The result of cell proliferation, apoptosis, migratory and invasion of Saos2 and U2OS after DSC2 was silenced by siRNA.** Cell Counting kit-8(CCK-8) assay showed that knocking down DSC2 inhibited the proliferation of Saos2 (P<0.001) and U2OS cells (P<0.001) relative to the control group and siRNA-negative control group(siNC). (^###^ P<0.001 in comparison to the control group. And, *** P<0.001 in comparison to the siNC group). (B) Cell apoptosis was detected by flow cytometry, and knockdown DSC2 could accelerate the apoptosis of Saos2 and U2OS cells (^##^ P < 0.01;^####^ P<0.0001 in comparison to the control group. And, ** P < 0.01; *** P<0.001 in comparison to the siNC group). The cell apoptosis of Saos2 and U2OS was decreased after DSC2 was silenced. (C) Wound healing assays were performed to evaluate migratory ability of Saos2 and U2OS cells in comparison to control group (^###^ P < 0.001) and siNC group (*** P<0.001), respectively. The ability of cell migratory of Saos2 and U2OS was decreased after DSC2 was silenced. (D) Transwell assays were performed to evaluate invasion ability of Saos2 and U2OS cells in comparison to control group (^###^ P < 0.001) and siNC group (*** P<0.001) respectively. The invasion ability of Saos2 and U2OS was decreased after DSC2 was silenced.

As shown in **Figure 9A**, the cell proliferation of Saos2 and U2OS was significantly decreased by knockdown DSC2. Besides, further research demonstrated that suppressing DSC2 could accelerate the apoptosis of OS cell (**Figure 9B**). The migration and invasion of U2OS and Saos2 cell were measured by wound healing and transwell assays. As shown in **Figure 9C** and **Figure 9D**, silencing DSC2 in the OS cells obviously restrained the cell migration and invasion.

## Discussion

The pathogenesis and progression mechanism of OS is extremly complex and elusive^17^. In the present study, via integration of single cell and bulk RNA sequencing, we preliminarily determined that DSC2 is high expressed in OS, which is a risk factor for survival and has a strong relationship with immune cell infiltration. What's more, in vitro experiments also demonstrated that DSC2 was high expressed in OS cells, and silencing DSC2 would suppress proliferation, migration and invasion of OS cells.

DSC2 is a key member of the desmosome family and takes a crucial part in initiating desmosome assembly^18^. Recent studies have found that increased expression of DSC2 in lung cancer is associated with lower overall survival and poor prognosis of patients. Furthermore, overexpression of DSC2 was found to promote tumor cell clustering, thereby improving cancer cells survival and metastasis^16^. In addition, DSC2 is up-regulated in triple negative breast cancer and is associated with early lymph node metastasis^19^. However, the study on the relationship between DSC2 and OS is extremely rare. Here, by scRNA seq data, we found that the expression level of DSC2 of OB cells from OS tissue was significantly higher than that from normal bone tissue. Additionally, DSC2 was upregulated in the OS tissues and cell lines contrasted to the normal counterparts from 9 GEO datasets. What's more, the mRNA and protein expression of DSC2 was significantly up-regulated in U2OS and Saos2 cell lines. Silencing DSC2 in the OS cells obviously restrained the cell migration and invasion. These results demonstrated that DSC2, a new oncogene gene, was crucial to the pathogenesis and progression in OS.

The prognosis of OS is very poor, and many prognostic models have been established. A current study established a prognostic model of OS base on the marker genes of tumor infiltrating lymphocytes^20^. Another study also indicated that marker gene derived from cuproptosis can be used to predict the prognosis of OS^21^. In the present, we found that DSC2 possessed a prominent prediction effect, which may provide a new target to improve the prognosis of OS.

Tumor immune microenvironment plays a key role in the occurrence and development of tumors^22^. Research have released that DSC2 was associated with immune cell infiltration in bladder cancer 23. Similarly, we also found that DSC2 was strongly associated with most immune cell infiltration, such as Neutrophils, M2 Macrophages, Eosinophils, resting Dendritic cells, CD8 T cells and CD4 memory activated T cells. Besides, 26 immune checkpoint genes were found to have a statistically significant positive correlation with DSC2.

This study has some limitations: 1) We found that DSC2 contributed to the development of OS in an oncogenic manner, but animal studies need to be conducted to verify our data. 2) The prognostic value of DCS2 should be further verified by immunohistochemistry.

## Conclusions

DSC2 may serve as an oncogene, which exert a crucial role in tumor progression, predicting prognosis and immune cell infiltration in OS.

## Supplementary Material

Supplementary figure.

## Figures and Tables

**Table 1:** Association between DSC2 expression and clinicopathological parameter in OS samples based on TARGET database.

Clinicopathological	Sample	DSC2 expression	P value
Parameters	N		
Age			0.064
<=17	66	2.28	
>17	19	2.01	
Gender			0.367
Female	37	2.15	
Male	48	2.28	
Tumor site			0.087
Arm/hand	6	2.14	
Leg/foot	77	2.22	
Pelvis	2	2.43	
Metastasis			0.053
Yes	21	2.07	
No	64	2.27	
