# Supplementary material for: Validation the role of desmocollin-2 in osteosarcoma based on single cell and bulk RNA seq and experimental analyses
Source: J Cancer. 2023 Aug 21;14(14):2619–32. doi: 10.7150/jca.87411 (PMC10539388; doi:10.7150/jca.87411)
Supplement: Supplementary file 1 — Supplementary figure. [file jcav14p2619s1.pdf]

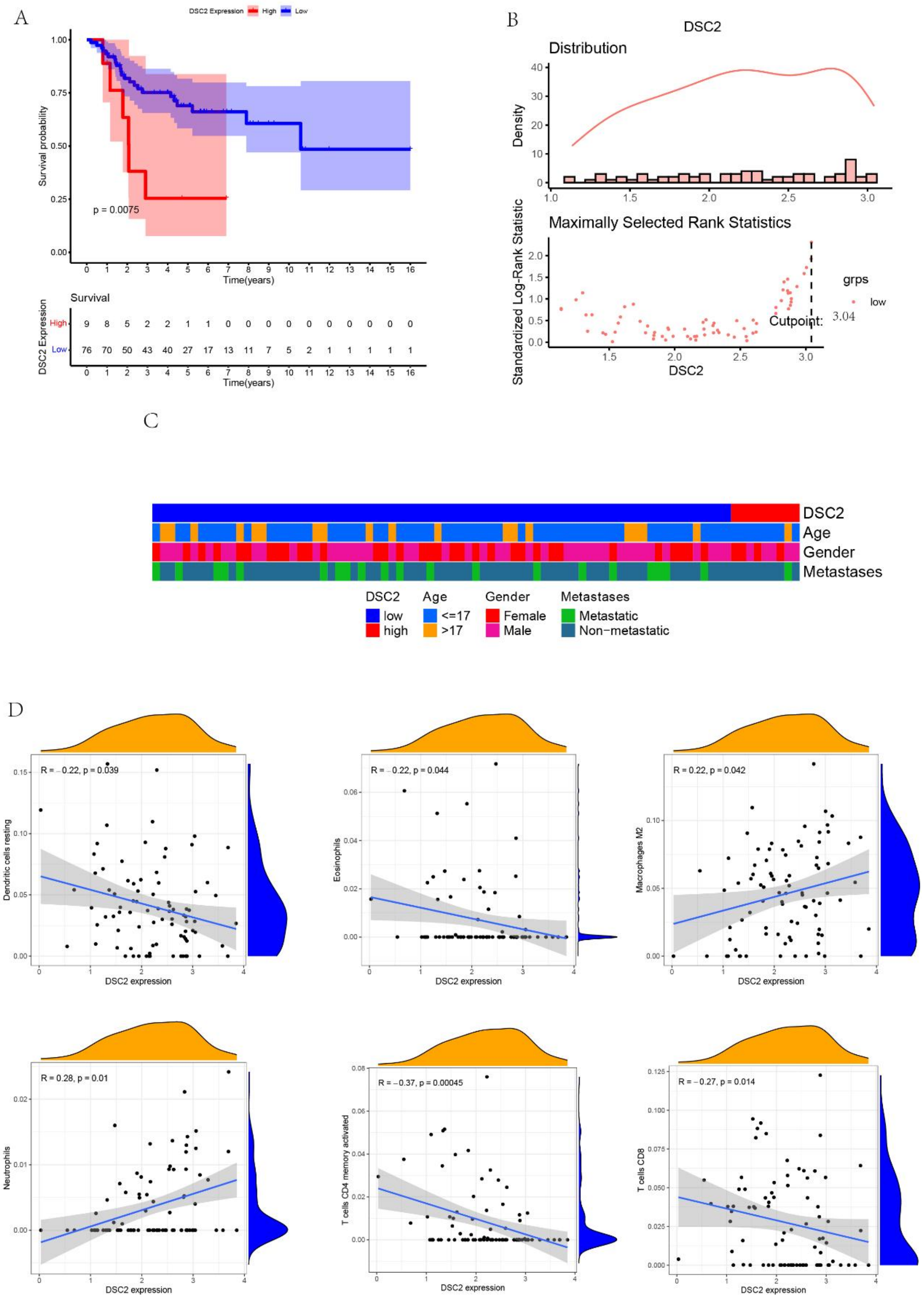

Supplementary Fig. 1 OS prognosis and immune cell infiltration associated with DSC2

(A) Kaplan-Meier analysis of DSC2 associated with fpkm values. (B)Identification of the optimal cutoff

value for the fpkm values. (C) the relationship among DSC2 expression and clinical phenotypes. (D) Immune cell infiltration associated with DSC2.
